# Association between arterial stiffness and risk of coronary artery disease

**DOI:** 10.12669/pjms.306.5584

**Published:** 2014

**Authors:** Ke-qin Luo, Xiao-wei Feng, Bing-can Xu, Hui-bao Long

**Affiliations:** 1 Ke-qin Luo, Department of Emergency, SunYat-Sen memorial Hospital, Sun Yat-Sen University, Guangzhou, 510120, China.; 2 Xiao-wei Feng, Department of Emergency, SunYat-Sen memorial Hospital, Sun Yat-Sen University, Guangzhou, 510120, China.; 3 Bing-can Xu, Department of Emergency, SunYat-Sen memorial Hospital, Sun Yat-Sen University, Guangzhou, 510120, China.; 4 Hui-bao Long. Department of Emergency, SunYat-Sen memorial Hospital, Sun Yat-Sen University, Guangzhou, 510120, China.

**Keywords:** Arterial stiffness, baPWV, cfPWV, Coronary Artery Disease

## Abstract

***Objective:*** To investigate the role of Brachial ankle Pulse Wave Relocity (baPWV) and cfPWV on the risk of Coronary artery disease and the interaction between baPWV and risk factors of Coronary artery disease (CAD).

***Methods:*** A case-control study was conducted at Department of Emergency, SunYat-Sen memorial Hospital, China. We collected 332 cases with coronary artery disease and 328 subjects without CAD between February 2012 and October 2013. A multivariate logistic regression analysis was performed to analyze the risk factors of CAD.

***Results:*** CAD subjects were more likely to be old age, and have higher BMI, waist-hip ratio, hypertension, fasting glucose, TG, carotid-femoral PWV (cfPWV) and baPWV, and CAD subjects had a lower TC, HDL–C and LDL-C. We found that older age, smoking, higher hypertension, TC, TG, HDL-C, LDL-C, carotid-femoral PWV (CfPWV) and baPWV were associated with risk of CAD. baPWV had significant interaction with age, TC, TG, HDL-C and LDL-C, carotid-femoral PWV (cfWV) was correlated with age, HDL-C and LDL-C.

***Conclusion: ***This study showed that baPWV and cfPWV are two independent factors for the risk of Coronary artery disease, and baPWV and cfPWV have interaction with age, TC, TG, HDL-C and LDL-C.

## INTRODUCTION

It is well known that arterial stiffness is an important risk factor for coronary artery disease (CVD) and arterial stiffness is caused by hypertension, end-stage renal disease and atherosclerosis.^1^^-^^3^ Detection of pulse wave velocity (PWV) is a non-invasive way to evaluate the arterial stiffness for atherosclerosis, and a significant association was found between PWV level and susceptibility of atherosclerotic disease.^4^^-^^6^ Recent studies have showed that PWV can be a predictive and prognostic factor for CVD.^6^^-^^8^

Assessing the PWV of arterial stiffness, carotid-femoral PWV (cfWV) is an important way to measure the stiffness of the thoracic and abdominal aorta. ingthe risk of CVD. It is reported that metabolic syndrome, cardiovascular disease, stroke and renal disease are all associated with increased baPWV.^9^^,^^10^

BaPWV included both the components of central and peripheral arterial stiffness, and it can influence the stiffness of large arteries. It is reported that baPWV and cfPWV can measure the arterial stiffness, and is associated with the susceptibility of CAD.^11^^,^^12^ However, few studies have investigated the interaction between baPWV and cfPWV and other potential risk factors of CAD, such as drinking, smoking, hypertension and diabetes as well as cholesterol. Therefore, we conducted a study to investigate the role of baPWV and cfPWV on the risk of CAD, and the interaction between baPWV and cfPWV and risk factors of CAD.

## METHODS

A case-control study was performed in Department of Emergency, SunYat-Sen memorial Hospital, China. All patients were diagnosed by angiographic evidence of ≥ 70% stenosis of one major coronary artery, or ≥ 50% stenosis of the left main coronary artery. The exclusion criteria were as follows: patients who were bedridden, mental illness and malignant tumors as well as severe systemic diseases. Initially, 356 patients with CAD were included between February 2012 and October 2013. Among them, 332 subjects provided blood samples for the testing of cardiac biomarkers, with a participation rate of 93.26%.

Three hundred fifty seven controls were included from population who came to our hospital for routine health check-up between February 2012 and October 2013. Control subjects who suffered from CAD or any other heart disease were excluded. Finally, 328 subjects were included in control group. All patients and control subjects signed a written informed consent Form. Our study was approved by the ethics committee of Department of Emergency, SunYat-Sen memorial Hospital.

The demographic and clinical characteristics were collected by a self-designed questionnaire. The demographic and clinical characteristics included sex, age, body mass index (BMI), waist-hip ratio, drinking and smoking status, hypertension, fasting glucose, Total cholesterol (TC), triglycerides (TG), low-density lipoprotein cholesterol (LDL-C), high-density lipoprotein cholesterol (HDL-C) levels. The investigation was performed by physicians or nurses in SunYat-Sen memorial Hospital. Waist-to-hip ratio (WHR) was calculated by measuring circumferences of the waist and hip, and BMI was calculated by measuring height and weight. The blood pressure was measured using a calibrated desktop sphygmomanometer after keeping in a supine position for ≥5 minutes.

The fasting glucose, total cholesterol (TC), triglycerides (TG), low-density lipoprotein cholesterol (LDL-C), high-density lipoprotein cholesterol (HDL-C) levels were measured using the enzymatic assays on an autoanalyzer (Roche Diagnostics, Indianapolis, IN, USA). 

The levels of cfPWV and baPWV were measured by blood pressure cuffs wrapped on the arm near the brachial artery and tibial artery of ankle, and evaluated using a volume-plethysmographic device (Omron Healthcare, Kyoto, Japan) after keeping in a supine position for ≥5 minutes. 

***Statistical analysis: ***Continuous variables are expressed by mean ± SD, and analyzed using a student t test. Categorical variables are expressed by n of subjects (%), and analyzed using a χ^2^-test. The odds ratios (OR) and corresponding 95% confidence intervals (CIs) were calculated by a multivariate logistic regression analysis and used to assess and evaluate the risk factor related to risk of CAD. Pearson correlation was performed to analyze the interaction between baPWV and cfPWV and risk factors of CAD. All P-values were two sided, and a *P*-value <0.05 was considered statistically significant. Statistical analysis was conducted using SPSS® version 16.0 (SPSS Inc., Chicago, IL, USA) for Windows®.

## RESULTS

The demographic and clinical characteristics of included cases and controls are shown in Table-I. The mean age of the enrolled CAD subjects and controls were 66.7±8.7 years and 63.5±12.3 years, respectively. There were 138 males in CAD patients and 150 males in controls. There were no significant differences in terms of sex and drinking habits between CAD patients and control subjects. CAD subjects were more likely to be old age, and have higher BMI, waist-hip ratio, hypertension, fasting glucose, TG, cfPWV and baPWV (P<0.05), and CAD subjects had a lower TC, HDL–C and LDL-C (P<0.05).

**Table-I:** The clinical characteristics of included subjects

**Characteristics**	**CAD patients**		**Controls**		***t *** **or χ** ^2^	***P*** **-value**
	**N=332**		**N=328**			
Age (years)	66.7±8.7		63.5±12.3		3.86	<0.001
Sex						
Male	138	41.57	150	45.73		
Female	194	58.43	178	54.27	1.16	0.28
BMI (kg/m^2^)						
<24	144	43.37	189	57.62		
>24	188	56.63	139	42.38	13.40	<0.001
Waist-hip ratio	0.90±0.12		0.85±0.10		5.81	<0.001
Smoking status						
Never	227	68.37	248	75.61		
Current or former	105	31.63	80	24.39	4.28	0.04
Drinking status						
Never	240	72.29	254	77.44		
Current or former	92	27.71	74	22.56	2.32	0.13
Hypertension						
No	124	37.35	152	46.34		
Yes	208	62.65	176	53.66	5.48	0.02
Fasting glucose (mmol/L)	5.56±0.08		5.32±0.07		41.00	<0.001
TC (mmol/L)	4.73±0.06		4.92±0.05		44.17	<0.001
TG (mmol/L)	2.03±0.09		1.82±0.07		33.48	<0.001
HDL–C (mmol/L)	1.34±0.13		1.47±0.11		13.87	<0.001
LDL-C (mmol/L)	2.52±0.24		3.03±0.14		33.29	<0.001
cfPWV (cm/s)	12.75±0.21		11.13±0.15		113.92	<0.001
baPWV (cm/s)	8.91±0.12		8.78±0.09		15.73	<0.001

TC: Total cholesterol; TG: triglycerides; HDL-C: low-density lipoprotein cholesterol;

HDL-C: high-density lipoprotein cholesterol.

The association between the clinical and demographic factors and risk of CAD was analyzed by multiple linear regression analysis and showed in Table-II. We found that older age, smoking, higher hypertension, TC, TG, HDL-C, LDL-C, CfPWV and baPWV were associated with risk of CAD, with ORs (CI) of 1.52(1.06-2.34), 1.52 (1.18-1.85), 0.74(0.53-0.91), 1.21(1.03-1.32), 0.71(0.52-0.92), 0.56(0.42-0.91), 1.28(1.03-1.35) and 1.12(1.03-1.27), respectively. However, no association was found between BMI, waist-hip ratio, drinking status and fasting glucose and risk of CAD (*P*>0.05).

**Table-II:** Association between risk factors and risk of CAD.

**Characteristics**	**Adjust OR**	**95% Confident Interval (CI)**	**P value**
Age	1.52	1.06-2.34	0.002
BMI	1.17	0.86-1.41	0.32
Waist-hip ratio	1.03	0.95-1.12	0.73
Smoking status	1.62	1.03-2.28	0.04
Drinking status	1.21	0.82-1.72	0.49
Hypertension	1.52	1.18-1.85	0.002
Fasting glucose	1.03	0.92-1.09	0.34
TC	0.74	0.53-0.91	0.005
TG	1.21	1.03-1.32	0.02
HDL-C	0.71	0.52-0.92	0.02
LDL-C	0.56	0.42-0.91	0.001
CfPWV	1.28	1.03-1.35	<0.001
baPWV	1.12	1.03-1.27	<0.001

We further analyzed the interaction of baPWV and CfPWV with risk factors of CAD (Table-III). We found that baPWV had significant interaction with age, TC, TG, HDL-C and LDL-C, and the Pearson’s coefficients were <0.001, 0.03, 0.02, 0.04 and 0.005, respectively. Moreover, cfPWV was correlated with age, HDL-C and LDL-C, with the Pearson’s coefficients of 0.02, 0.02 and 0.03, respectively (P>0.05).

**Table-III:** Pearson correlation of baPWV with other risk factors of CAD.

**Characteristics**	**baPWV**		**cfPWV**	
	**Pearson’s coefficient**	***P*** ** value**	**Pearson’s coefficient**	***P*** ** value**
Age	0.192	<0.001	0.11	0.02
Smoking status	0.027	0.36	0.013	0.53
Hypertension	0.12	0.42	0.092	0.65
TC	0.025	0.03	0.011	0.29
TG	0.028	0.02	0.017	0.08
HDL-C	0.16	0.04	0.06	0.02
LDL-C	0.082	0.005	0.051	0.03

## DISCUSSION

Increased vascular stiffness is independently associated with outcome of cardiovascular events, but the underlying mechanisms of cardiovascular events is not completely understood.^13^ In this study, we showed that baPWV and cfPWV was independently correlated with risk of CAD, and older age, smoking, higher hypertension, TC, TG, HDL-C, LDL-C, CfPWV and baPWV were associated with risk of CAD. Moreover, we found that baPWV had significant interaction with age, TC, TG, HDL-C and LDL-C, and cfPWV was correlated with age, HDL-C and LDL-C on the risk of CAD.

It is known that increased arterial stiffness hinders the hemodynamic buffering effect for the cardiovascular system, which causes the increased systolic blood pressure and pulse pressure, coronary arterial disease and left ventricular hypertrophy.^14^^,^^15^ Therefore, measuring aortic PWV can be used as a better method to evaluate the aortic stiffness in assessing subclinical target organ damage.^16^^,^^17^ Several previous studies have investigated the association between baPWV level and risk of cardiovascular events.^18^^-^^21^ Chae et al. showed that baPWV was an independent predictor of the risk of CAD, but it has a limited value for predicting the severity of CAD in patients with chest pain.^18^ Han reported that increased baPWV was associated with risk of cardiovascular events, especially for ischemic stroke.^19^ A recent study showed that baPWV was associated with risk of CAD, and baPWV had significantly correlation with BMI, SBP, DBP, TC, TG, HDL-C and LDL-C.^20^ For cfPWV level, two studies reported the association between cfPWV level and risk of CAD.^22^^,^^23^ Tanaka et al. reported that cfPWV and baPWV were predictors for arterial stiffness, and the two factors showed similar extent of associations with cardiovascular disease related risk factors and clinical events.^22^

The association between baPWV and cfPWV and CAD risk can be explained by several mechanisms. Arterial stiffness causes premature return of the refulected pulse wave in later systole, and causes increased central pulse pressure and load on the left ventricle, and thus reduces ejection fraction and enhances myocardial oxygen demand.^24^ The decreased absorption capacity of the arterial wall can cause wall injury, and accelerate the progression of atherosclerosis.^25^ Previous studies have showed that baPWV and cfPWV were closely associated with CAD, suggesting that baPWV and cfPWV are two predictor for central arterial stiffness.^18^^-^^20^^,^^22^^,^^23^

Our study showed that baPWV had significant interaction with age, TC, TG, HDL-C and LDL-C, which is in concordance with previous studies.^20^^,^^26^^,^^27^ Urbina et al. reported that baPWV was correlated with blood pressure.^26^ Another study showed that blood pressure was associated with baPWV in young adults.^27^ Zhu et al. reported baPWV was significantly associated with age, BMI, TC, TG, HDL-C and LDL-C.^20^ Moreover, we also found cfPWV was correlated with age, HDL-C and LDL-C. One previous study showed that LDL-C and HDL-C were associated with cfPWV.^28^

***Limitations of the study:*** First, the present study is hospital-based case-control study, and control subjects who participated in our study came for routine health check-up, these patients may focus on their health. Therefore, selection bias may be there. Second, the sample size is relatively small in our study. The small sample size may reduce the statistical power. Therefore, further large sample studies are greatly needed to confirm the association between baPWV and cfPWV and CAD risk.

In conclusion, our study shows that baPWV and cfPWV are two independent factors for the risk of CAD, and baPWV and cfPWV have interaction with age, TC, TG, HDL-C and LDL-C. Therefore, baPWV and cfPWV can be a screening tool for detecting patients with higher risk of CAD.

## Authors Contributions:

**KQL & XWF:** Designed and performed the study, did statistical analysis & editing of manuscript.

**BCX & HBL:** Did data collection and manuscript writing.
